# The Biochemical Role of Bitter Melon (*Momordica charantia* L.) Aqueous Extract in Regulating Hepatic Gluconeogenesis in Rats Fed Ketogenic Diet

**DOI:** 10.1002/fsn3.71457

**Published:** 2026-02-10

**Authors:** Ghalia Shamlan

**Affiliations:** ^1^ Department of Food Sciences and Nutrition, College of Food and Agricultural Sciences King Saud University Riyadh Saudi Arabia

**Keywords:** bitter melon aqueous extract, gluconeogenesis, ketogenic diet, ketone bodies, triglycerides

## Abstract

The ketogenic diet (KD) has been suggested as a useful lifestyle intervention for metabolic syndrome; however, its long‐term metabolic effects remain debated. This study evaluated the biochemical impact of supplementing KD‐fed rats with a diluted aqueous extract of Bitter Melon (BM) on the regulation of hepatic gluconeogenesis. Thirty‐two male rats were divided into four groups: G1 (Control), G2 (KD), G3 (BM extract, 1.5 g/kg body weight), and G4 (KD + BM extract, 1.5 g/kg body weight). Serum and liver samples were analyzed for biochemical parameters. KD reduced glucose, insulin, triglycerides (TG), Free Fatty Acids (FFA), glycogen, pyruvate carboxylase, and PEPCK, while elevating cholesterol (+34%), lactate, and ketone bodies (*p* < 0.05). BM supplementation partially restored glucose (+15%), insulin, TG, FFA, glycogen, and enzyme activity, while lowering cholesterol (−14%), lactate (−16%), and ketone bodies (−27%) (*p* < 0.05). Histological analysis confirmed improved liver architecture in BM‐treated groups. BM aqueous extract counteracts KD‐induced metabolic disturbances, improving glucose and lipid homeostasis and supporting its role as a safe adjunct to KD for long‐term metabolic management.

## Graphical abstract

Metabolic effects of a ketogenic diet and bitter melon extract on hepatic gluconeogenesis and glucose–lipid homeostasis.

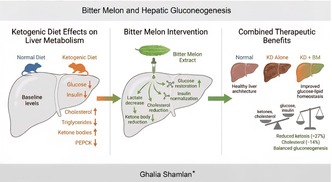

## 1 Introduction

A diet that is richer in fat than in protein and carbohydrate, or that completely carbohydrate rich foods (e.g., bread, sugar, grains) is known as a ketogenic diet (KD). Such a diet leads to reduced carbohydrate and protein metabolism but heightened fat metabolism, moreover, inducing changes in the overall metabolism of the body by producing fat‐derived ketone bodies (acetoacetate and β‐hydroxybutyrate) and lowering the level of glucose in blood (Castellana et al. 2020). It has been suggested that a number of metabolic diseases can be successfully treated with KD, such as epilepsy, diabetes, and tumor development (Kumar et al. 2021; Neth et al. 2020).

There are sufficient researches on the positive effect of KD on metabolism (Charlot and Zoll 2022). For instance, Zhao et al. (2012) reported that, KD improved athletic performance and improper motor function in a model of amyotrophic lateral sclerosis. Choi et al. (2016) claimed that, KD could be a treatment option for neurological disorders, since ketone bodies serve as the primary source of energy for brain instead of glucose (Choi et al. 2016). Volek et al. (2016) explored the role of ketone bodies as potent signaling molecules in the brain, helping cells to adapt to fasting, physical activity, dietary interventions, and other environmental stimuli (Volek et al. 2016).

On the other hand, the implications of KD, including limited food range available, lack of macronutrient profile balance, and gastrointestinal adverse effects, can make it difficult to follow this diet long term (Andrewski et al. 2022; Ludwig 2020; Paoli et al. 2015). Consequently, supplements that replicate the ketosis attained via KD or protracted food deprivation have been developed (Gutiérrez‐Repiso et al. 2019). Therefore, two distinct types of ketosis have been distinguished, namely, nutritional or endogenous ketosis produced by KD compliance and peripheral or exogenous ketosis produced by dietary supplements (Shaw et al. 2020). A study by Kesl et al. (2016) revealed that, ketone supplementation caused rapid and sustained elevation of blood ketones (1–5 mmol/L β‐hydroxybutyrate) for hours after oral administration (Kesl et al. 2016).

In mammalian organisms, glucose supply to the brain, production of anaerobic energy, and glucose supply to cells for biosynthesis are all critically dependent on glucose synthesis (Rask‐Madsen and Kahn 2012). This process is even more important under conditions of limited food intake and exhaustion of glycogen reserves in liver (Schutz et al. 2021). It was noticed that, 2 h of fasting could deplete the hepatic glycogen reserves, and subsequently gluconeogenic process from amino acids and other non‐glycosidic reserves becomes essential for ensuring glucose supply (Yang et al. 2015).

The liver is a multifunctional organ accountable for detoxification and macronutrients metabolism (Graffmann et al. 2020). Hepatocytes are a place of bile synthesis that is essential for digestion and absorption of lipids (Hundt et al. 2017). As well, anabolic and catabolic transformations of fatty acids, triacylglycerols and sphingolipids, also ketogenesis processes occur in liver (Lepionka et al. 2021; Nguyen et al. 2008).

BM ( *Momordica charantia* L.) is a member of the Cucurbitaceae family is a climber and its fruits are widely used as medicinal foods in numerous countries (e.g., China, India, Malaya, Thailand) to treatment of hyperlipidaemia and diabetes (Zheng et al. 2020). It is herbaceous plant that can reach a height of about 5 m and its with leaves of 4–12 cm in size with 3–7 cm distinctly separate lobes (Rahman, Kamrunnahar, et al. 2021; Saeed et al. 2021; Tan et al. 2016). It looks as a small cucumber, as it typically displays a rectangular and oblong shape, also it has a pulp and sizable flat seeds enveloping a relatively thin flesh layer (Saeed et al. 2011). In terms of nutrition, BM consists largely of water (91.8%), with smaller proportions of fat (0.20%), carbohydrate (4.2%), and fiber (1.4%) (Hadi et al. 2022). Its seeds have a content of 35%–40% oil, 3.33% Monounsaturated Fatty Acids (MUFA), and 36.71% Saturated Fatty Acids (SFA). Moreover, BM has the greatest proportion of Polyunsaturated Fatty Acids (PUFA) (59.96%) (Hadi et al. 2022). One PUFA that is especially significant is the conjugated linolenic acid called α‐eleostearic acid (54.26%) (Saeed et al. 2018; Yeh et al. 2003). In addition, BM has high levels of polysaccharides, saponins, polyphenols, and polypeptide (Gao et al. 2021). The BM Polysaccharides (BPS) have been suggested to have great potential as evidence indicates their physiological roles, including enhanced modulation of immunity, targeting of oxidants, and decreasing sugar levels in the blood (Bai et al. 2018).

The two major uses BM fruits are in cooking in countries such as Bangladesh and India, and in traditional medicine especially in developing nations like Brazil, China, Colombia, Cuba, Ghana, Haiti, India, Mexico, Malaya, Nicaragua, Panama, Peru, and Bangladesh (Alam et al. 2015). Diabetes is the disease most widely treated with BM, followed by another diseases including dysmenorrhea, eczema, gout, jaundice, kidney (stone), leprosy, leukorrhea, piles, pneumonia, psoriasis, rheumatism, and scabies (Alam et al. 2015; Muronga et al. 2021).

A major therapeutic effect is the hypolipidemic effect, showing BPS with effective lipid‐lowering properties, although complete understanding of the action mechanism was not be achieved yet (Shen et al. 2018).

Sinha and Jastreboff (2013) conducted a systematic review of the various herbal products typically used to manage Type 2 Diabetes (T2D) and provided evidence that, BM could indeed reduce the levels of glucose in the blood. Insulin‐like peptides, alkaloids, and charantins have been identified as the main *M. charantia* compounds that can lower blood glucose (Sinha and Jastreboff 2013). Han et al. (2018) proposed that, hypoglycaemic action was exhibited by triterpenoids as well, although evidentiary support for this is currently limited (Han et al. 2018). Meanwhile, according to the findings of mechanistic research, the anti‐inflammatory action of BM is the source of its effect against obesity (Juan et al. 2017). BM has also been suggested to have hypoglycaemic, antipyretic, antibacterial, antihelminthic, antimalarial, antiulcerogenic, and immunomodulatory effects (Fuangchan et al. 2011; Upadhyay et al. 2015).

The present study was designed to estimate the biochemical role of BM aqueous extract ( *M. charantia* L.) supplementation on the regulation of gluconeogenesis in rats fed KD.

## 2 Materials and Methods

### 2.1 GC–MS Analysis of the Extract

The GC–MS profile of the bitter melon ( *M. charantia* ) fruit 90% methanol extract was analyzed using an Agilent gas chromatography (GC; 7890A Agilent Technologies, Santa Clara, CA, USA) coupled with an inert mass spectrometer (5975C; MS). The system was fitted with a DB‐5MS GC column (30 m × 0.25 mm inner diameter, 0.25 μm), a Triple‐Axis detector, and a 7693 automated liquid sampler. The system parameters were: 1 μL injection volume; 280°C injection temperature; 300°C column temperature; He carrier gas with a flow rate of 1 mL/min; 70 eV ionization energy.

### 2.2 Preparation of Aqueous Extract of BM

As per the approach outlined by Jayasooriya et al. (2000). BM ( *M. charantia* L.) fruits used in this study were authenticated, and a voucher specimen (Voucher No. BM 968) was deposited. In this study, the pulp (edible flesh) of fresh bitter melon ( *M. charantia* L.) fruits was used. The fruits were cleaned, peeled, and only the pulp was chopped, freeze‐dried, powdered, and prepared as aqueous extract. A portion of the dried material was weighed, and water was added at 80°C to obtain 1% extract (w/v) of fresh extract. This process was repeated daily. Once the extract had reached room temperature, it was filtered and given to the animals as a *ad libitum* 10 min after (Jayasooriya et al. 2000).

### 2.3 Experimental Diet Content

AIN‐93 according to Reeves et al. (1993) was followed in the formulation of every diet. Butter fat was used and substituted the corn starch (69 g of butter fat was used/100 g diet).

### 2.4 Experimental Animals

A total of 32 Sprague–Dawley male Albino rats weighing 100–120 g were equally divided into four groups. The rats were properly cared for and kept separately in cages of stainless steel and with bottoms of wire mesh under conditions of 25°C temperature, 50% humidity, and light–dark cycle. No restrictions were put on access to food and water. Ethical confirmation was approved by Standing Committee for Scientific Research—Jazan University, Saudi Arabia (REC‐45/07/968).

### 2.5 Experimental Design

- Group (1): control animals received distilled water and standard diet,
- Group (2): KD; group were administered distilled water and KD,
- Group (3): normal rats received oral dose of BM extract (1.5 g/kg body weight) according to Abas et al. (2014) daily for 6 weeks.
- Group (4): rats fed ketogenic diet and received oral dose of BM extract (1.5 g/kg body weight) according to Abas et al. (2014) daily for 6 weeks.

### 2.6 Blood Analysis

When the experiment duration finished, the rats were fasted overnight prior to blood collection and were then sacrificed under ether anesthesia. Blood was collected by retro‐orbital puncture. Serum separation involved leaving the blood samples to coagulate for half an hour at 25°C and 20‐min centrifugation at 3000 rpm. Several aliquots were subsequently created, which were placed in storage at −20°C to await use. Extraction and saline washing of the liver were also performed.

### 2.7 Serum Analysis

An enzymatic colorimetric procedure kit (Kamiya Biomedical Co., CA, USA) was employed to detect the fasting glucose level in fresh serum, while commercial kits (Stanbio, Boerne, TX, USA) were employed for spectrophotometric measurement of the levels of β‐hydroxybutyrate and acetoacetate. Furthermore, Biovision enzymatic colorimetric kits were used to determine the levels of total cholesterol and triacylglycerols in the serum. Radioimmunoassay (Kamiya Biomedical Co., CA, USA) was conducted to measure the insulin level, whilst an enzymatic kit (Biovision Kit, CA) was used to detect the levels of Free Fatty Acids (FFA).

### 2.8 Liver Analysis

Liver samples were separated, rinsed, and washed in saline solution (NaCl 0.9%), followed by blotting on filter paper. The liver was further subjected to quick freeze‐clamping and stored at −20°C for glycogen and enzyme analysis. According to Bruss and Black (1978), glycogen measurement involved fast excision of 0.5–1.0 g of liver and digestion in 30% (W/v) KOH. While, the procedure proposed by Baly et al. (1985) was adopted to detect the liver homogenates of pyruvate carboxylase and phosphoenolpyruvate carboxykinase.

### 2.9 Statistical Analysis

The SPSS software (version 22.0) was used for the statistical data analysis. Expression of the data took the form of mean ± SD. *t*‐Test was conducted to determine inter‐group statistical differences, which were supposed to be of significance when *p* < 0.05.

### 2.10 Histological Measurements

Hematoxylin and eosin staining was applied after sectioning, deparaffinizing, and storing liver tissue samples from each group in a 10% formalin solution, which were evaluated by a pathologist.

## 3 Results

### 3.1 GC–MS Profile of Bitter Melon Fruit Extract

**FIGURE 1:**
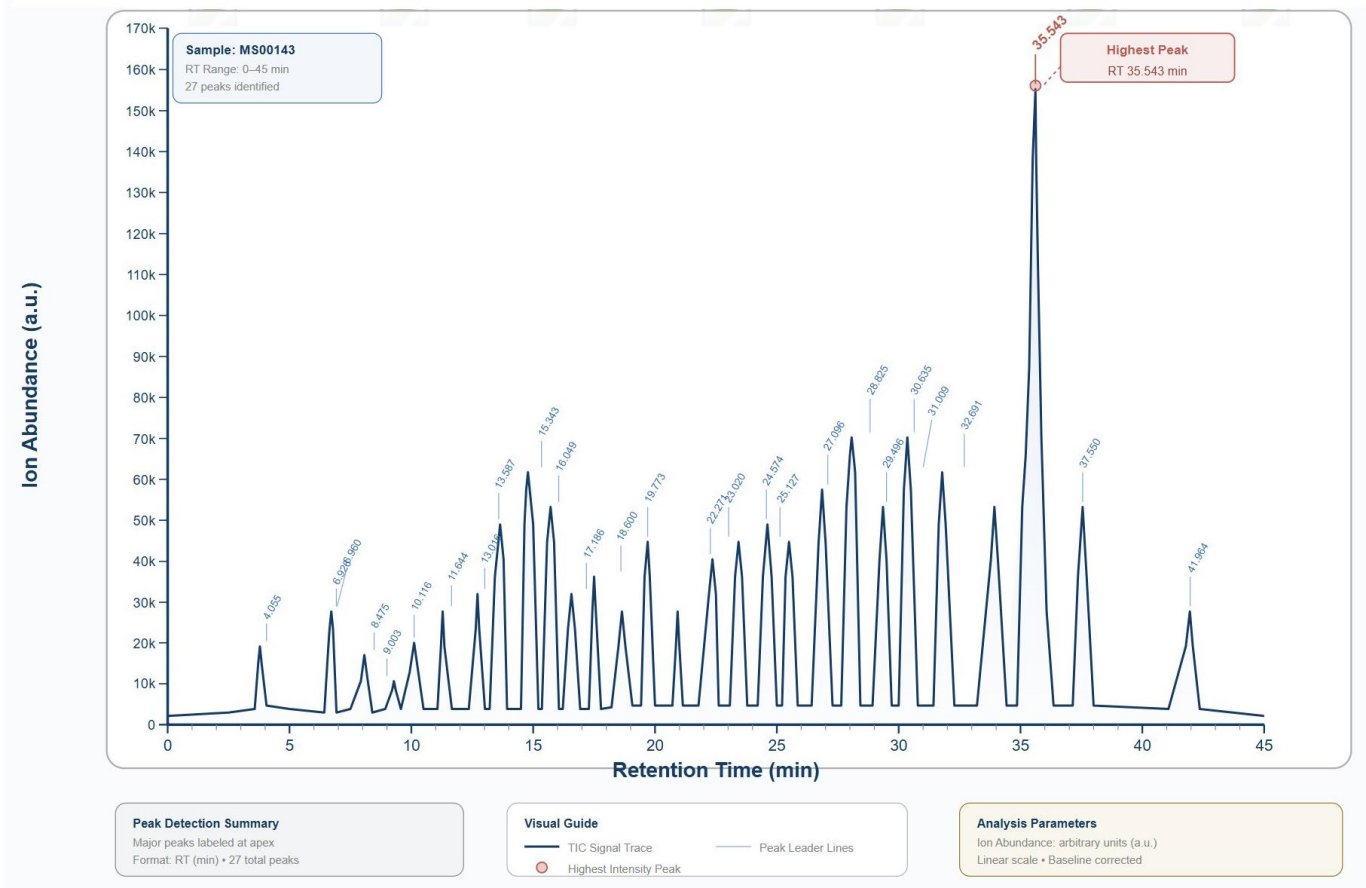
GC–MS profile of bitter melon fruit methanol extract.

GC–MS analysis of bitter melon fruit methanol extract exhibited bioactive substances (Table 1; Figure 1) in considerable amounts, such as morpholine‐4‐carboxylic acid (9.96% of the total peak area), 1,2‐cyclopentanedione (7.90% of the total peak area), 7,9‐di‐tert‐butyl‐1‐oxaspiro (4,5)deca‐6,9‐diene‐2,8‐dione (7.77% of the total peak area), and diethyl phthalate (6.16% of the total peak area).

**TABLE 1:** GC–MS compounds of bitter melon fruit methanol extract.

No.	RT	Peak area (%)	Compound name	Molecular formula/molecular weight (g/mol)	Compound nature	Bioactivity
1	6.93	9.70	1,2‐Cyclopentanedione	C_5_H_6_O_2_; 98.1	Cyclic diketone	Not reported
2	8.63	3.39	1,3‐Cyclohexanedione	C_6_H_8_O_2_; 112.13	Cyclic diketone	Not reported
3	13.02	1.66	Octanoic acid	C_8_H_16_O_2_; 144.21	Fatty acid	It has antibiotic features against *Escherichia coli*, *Staphylococcus aureus* , and *Candida albicans* (Guo et al. 2006; Nair et al. 2005; de Solis los Santos et al. 2009; Zhang et al. 2019)
4	15.34	4.29	Nonanoic acid	C_9_H_18_O_2_; 158.24	Fatty acid	It has antifungal properties (Chadeganipour and Haims 2001; Chung et al. 2011; Davis et al. 1997; Månsson et al. 2005)
5	16.65	0.58	Piperidine	C_5_H_11_N; 85.15	Heteromonocyclic compound	Its derivatives exhibit antioxidant, antipsychotic, antidepressant, antidiabetic, anticancer, anti‐inflammatory, analgesic, antibacterial, antimalarial, and antifungal properties (Abdelshaheed et al. 2021)
6	17.45	3.59	Butanoic acid, 4‐chloro‐3‐oxo‐, methyl ester	C_5_H_7_ClO_3_; 150.56	Keto carboxylic acid	Not reported
7	19.77	2.41	Cycloheptasiloxane, tetradecamethyl—	C_14_H_42_O_7_Si_7_; 519.07	Macrocyclic organosiloxane	It has antibacterial, immunomodulatory, antitumor, antifungal, and antifouling activities (Lee et al. 2024)
8	22.27	6.16	Diethyl phthalate	C_12_H_14_O_4_; 222.24	Benzenedicarboxylic acid	Phthalic acid esters have allelopathic, antimicrobial, and insecticidal properties (Huang et al. 2021)
9	24.57	1.93	2‐Octyl benzoate	C_15_H_22_O_2_; 234.33	Phenyl ester	Not reported
10	27.24	4.01	1,2‐Benzenedicarboxylic acid, bis (2‐methylpropyl) ester	C_16_H_22_O_4_; 278.34	Phthalate ester	It possesses chemopreventive activities against osteosarcoma cells (Kumar et al. 2022)
11	28.88	7.77	7,9‐Di‐tert‐butyl‐1‐oxaspiro (4,5) deca‐6,9‐diene‐2,8‐dione	C_17_H_24_O_3_; 276.4	Cyclic ketone	Not reported
12	32.93	9.96	Morpholine‐4‐carboxylic acid	C_13_H_18_N_4_O_4_S; 326.4	Carboxylic acid derivative	Not reported

### 3.2 Effect of Different Treatments on Serum Levels of Insulin, Glucose, Triglycerides, and Total Cholesterol Levels

Effect of different treatments on serum levels of glucose, insulin, TG, total cholesterol and FFA was represented in Table 2, results showed that, KD reduced levels of glucose, insulin, TG and FFA, while total cholesterol level was significantly elevated as compared to control one. While, supplementation with BM extract improved these parameters. Moreover, it was noticed that supplementing BM extract to normal rat induced a slight reduction in glucose and FFA levels as compared to control rats, but no significant difference was noticed regarding the other parameters.

**TABLE 2:** Effect of different treatments on serum levels of insulin, glucose, triglycerides, and total cholesterol levels.

Parameter	Control (G1)	Ketogenic diet (G2)	Bitter melon (BM) extract (G3)	Ketogenic diet+ bitter melon (BM) extract (G4)
Glucose (mg/dL)	85.62 ± 1.6	54.86 ± 2.3^a^	71.92 ± 1.8^a^, ^b^	62.94 ± 1.4^a^, ^b^
Insulin (U/L)	7.51 ± 0.14	0.7 ± 0.93^a^	7.62 ± 0.26^b^	5.25 ± 0.24^a^, ^b^
Triglycerides (TG) (mg/dL)	64.43 ± 2.86	43.65 ± 1.65^a^	63.74 ± 2.88^b^	45.87 ± 1.87^a^, ^b^
Total cholesterol (mg/dL)	76.73 ± 2.75	102.64 ± 2.88^a^	78.93 ± 2.55^b^	88.23 ± 2.87^a^, ^b^
Free fatty acids (FFA)	0.55 ± 0.02	0.27 ± 0.73^a^	0.42 ± 0.65^a^, ^b^	0.37 ± 0.73^a^, ^b^

^a^ Significant to control.

^b^ Significant to ketogenic group.

### 3.3 Effect of Different Treatments on Serum Levels of Ketone Bodies (β‐Hydroxy Butyrate, Acetoacetate) and Lactate

The effect of different treatments on serum levels of ketone bodies (β‐hydroxy butyrate, Acetoacetate) and Lactate, was shown in Table 3. However, in comparison to control rats, KD caused a significant elevation in serum level of β‐hydroxy butyrate, acetoacetate and Lactate. Similarly, supplementing BM extract improved these parameters. Results also noticed that, supplementing BM extract to normal control rats has no effect on levels of β‐hydroxy butyrate, acetoacetate while it causes a slight reduction in lactate level as compared to control rats.

**TABLE 3:** Effect of different treatments on Serum levels of ketone bodies (β‐hydroxy butyrate, acetoacetate) and Lactate.

Parameter	Control (G1)	Ketogenic diet (G2)	Bitter melon (BM) extract (G3)	Ketogenic diet+ bitter melon (BM) extract (G4)
β‐hydroxy butyrate	74.65 ± 2.65	152.65 ± 4.4^a^	70.53 ± 4.97^b^	110.86 ± 2.55^a^, ^b^
Acetoacetate	47.83 ± 1.54	62.87 ± 2.65^a^	44.21 ± 3.54^b^	50.82 ± 2.74^a^, ^b^
Lactate	1.54 ± 0.73	2.53 ± 0.67^a^	1.44 ± 0.43^a^, ^b^	2.12 ± 0.77^a^, ^b^

^a^ Significant to control.

^b^ Significant to ketogenic group.

### 3.4 The Effect of Different Treatments on Metabolic Measurements: Glycogen, Pyruvate Carboxylase and Phosphoenolpyruvate Carboxykinase Contents in Liver Homogenate

**FIGURE 2:**
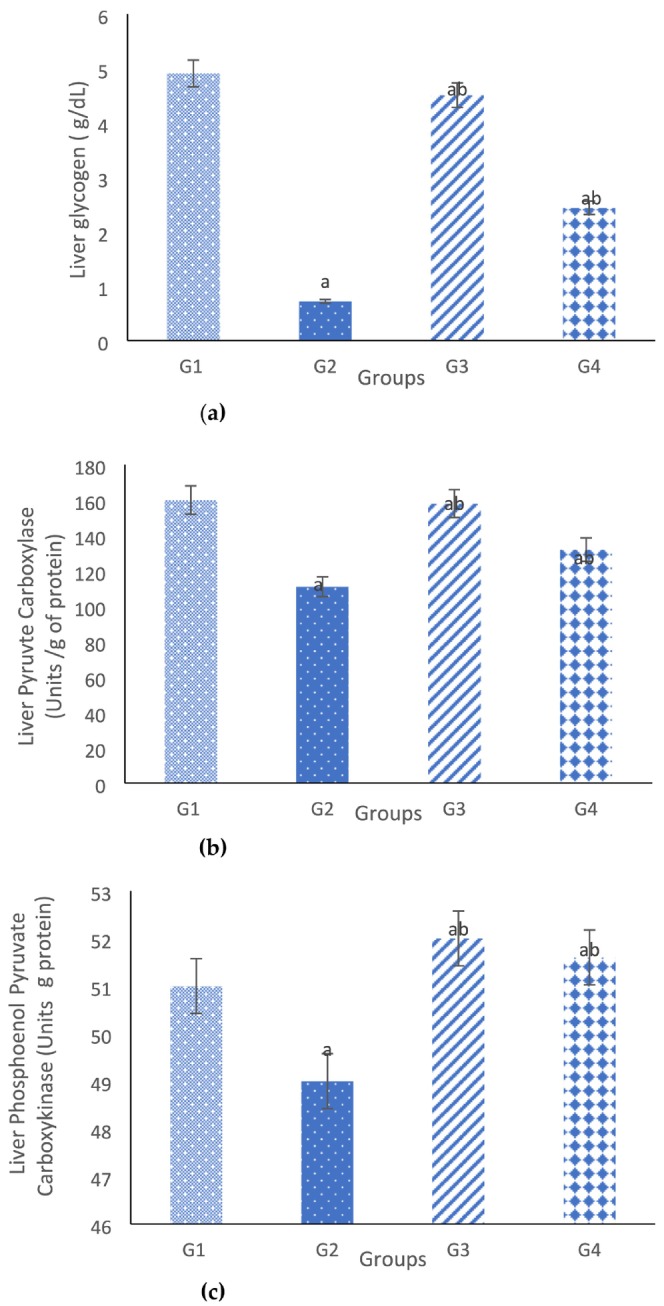
Represents the effect of different treatments on metabolic measurements: glycogen, pyruvate carboxylase, and phosphoenolpyruvate carboxykinase contents in liver homogenate. G1: Control group; G2: Ketogenic diet; G3: Bitter melon (BM) extract group; G4: Ketogenic diet + Bitter melon (BM) extract. (a) Significant to control. (b) Significant to ketogenic group (c) significantly different from Bitter melon alone and from the combined treatment.

Results in Figure 2a–c showed the effect of different treatments on liver homogenate level of glycogen, pyruvate carboxylase and phosphoenolpyruvate carboxylase respectively. KD was able to reduce these parameters significantly as compared to normal control diet. Conversely, supplementation of BM extract induced an improvement in these parameters.

### 3.5 Histological Findings of Rat Livers, Stained With Hematoxylin–Eosin (40×) Magnification

**FIGURE 3:**
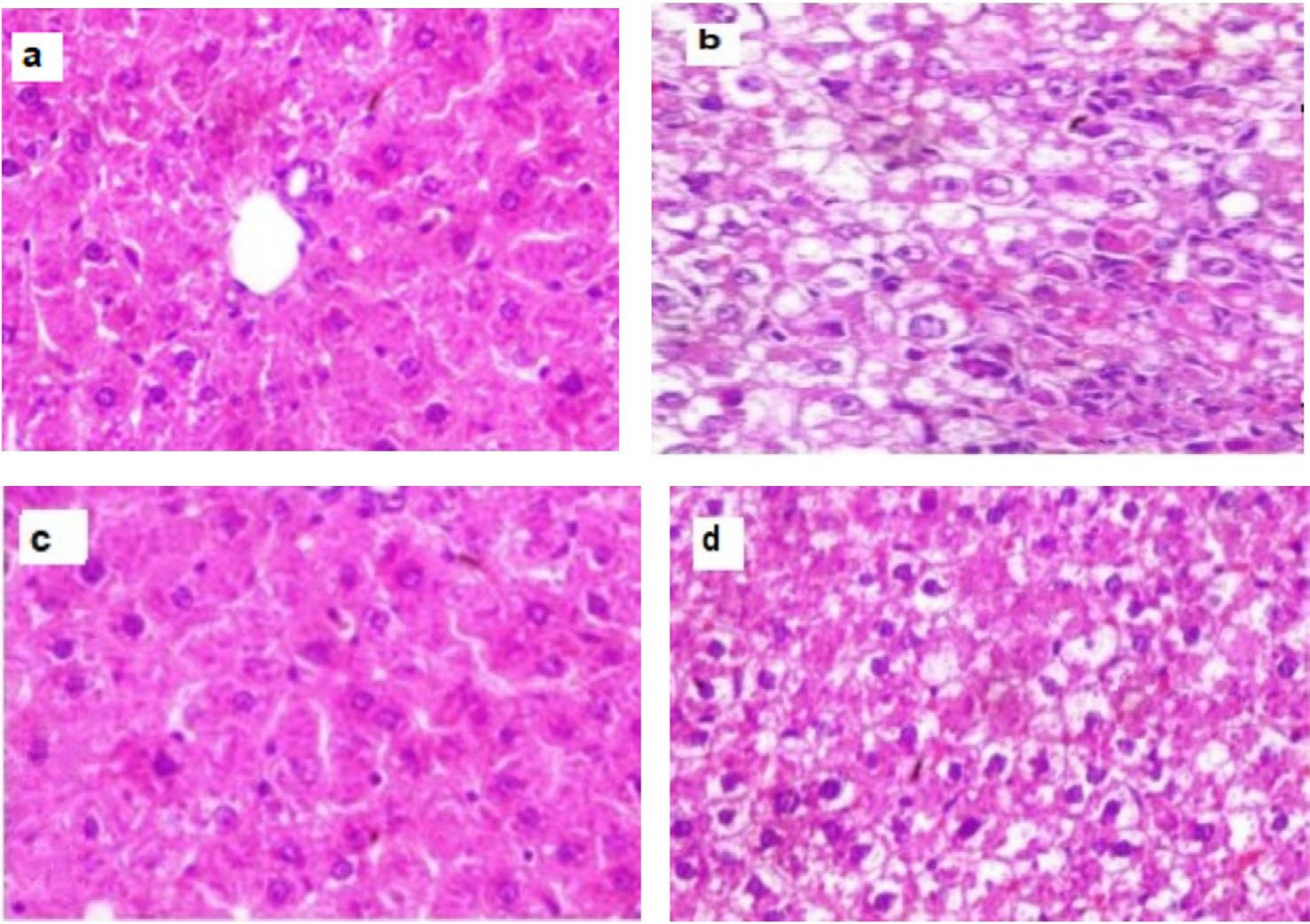
Histological findings of rat livers, stained with hematoxylin–eosin (40×) magnification. (a) Control group show normal liver architecture; the hepatic lobule shows the central vein; (b) Ketogenic diet lipid droplets were accumulated in cytoplasm; (c) Bitter melon (BM) extract group shows normal liver architecture; (d) Ketogenic diet + Bitter melon (BM), an improvement in liver texture was recognized.

Results of histological examination were determined in Figure 3, it was detected that both control rat group and rat group fed BM extract (Figures 2b and 3a) respectively, showed a normal liver architecture, while lipid droplets were accumulated in cytoplasm of rat liver in KD group (Figure 3c), on the other hand, supplementing BM extract to rats fed KD improved liver texture, while less lipid droplets were recognized (Figure 3d).

## 4 Discussion

Most of bitter melon fruit substances did not demonstrate biological activities, except for diethyl phthalate, which was found to possess esters with allelopathic, antimicrobial, and insecticidal properties (Huang et al. 2021). However, other substances with lower percentages showed biological properties. For example, nonanoic acid (4.29%) and octanoic acid (1.66%) demonstrated antibacterial and antifungal activities (Guo et al. 2006; Nair et al. 2005; de Solis los Santos et al. 2009; Zhang et al. 2019). Cycloheptasiloxane, tetradecamethyl‐ (2.41%) illustrated immunomodulatory, antitumor, antifungal, antibacterial, and antifouling properties (Lee et al. 2024). Furthermore, 1,2‐benzenedicarboxylic acid, bis (2‐methylpropyl) ester showed chemopreventive properties against osteosarcoma cells (Kumar et al. 2022), while derivatives of piperidine (0.58%) were reported to exert antibacterial, antimalarial, antifungal, anti‐inflammatory, analgesic, antioxidant, antipsychotic, antidepressant, antidiabetic, and anticancer properties (Abdelshaheed et al. 2021). In this study, the aqueous extract of the pulp of *M. charantia* L. was prioritized in the KD, as it contains bioactive compounds such as polysaccharides, saponins, polyphenols, and triterpenoids that were expected to counteract KD‐induced metabolic alterations (Bara et al. 2025).

Hepatic glycogenolysis maintains blood glucose after feeding, while gluconeogenesis sustains it during fasting. Glucose homeostasis relies on insulin for uptake in the fed state and glucagon with counter‐regulatory hormones for hepatic release during fasting (Sasaki et al. 2017; Vallon 2020). Glucose homeostasis both consumes and produces glucose; during fasting, hepatic gluconeogenesis is essential to maintain blood glucose in KD‐fed rats (Hatting et al. 2018; Rahman, Hossain, et al. 2021).

Rats administered KD displayed fasting serum glucose that was indicative of severe hypoglycaemia, meaning that, the possibility of gluconeogenesis was reduced as Yang et al. (2015). The decline in serum glucose, insulin, TG, FFA, and liver glycogen, pyruvate carboxylase, and phosphoenolpyruvate carboxykinase observed in KD‐fed rats was attributed to carbohydrate restriction, glycogen depletion, reduced insulin secretion, enhanced lipolysis and β‐oxidation, and down‐regulation of gluconeogenic enzymes (Ahmad et al. 2024; Effinger et al. 2023). Conversely, the increases in total cholesterol, lactate, and ketone bodies (β‐hydroxybutyrate and acetoacetate) in KD‐fed rats were attributed to enhanced hepatic cholesterol synthesis under high‐fat intake, increased lactate production due to glycogen depletion and reliance on anaerobic glycolysis, and accelerated ketogenesis from fatty acid β‐oxidation in the absence of sufficient carbohydrate supply (Ahmad et al. 2024; Yang et al. 2015) argued that, lack of maintenance of normal blood glucose during fasting or KD administration for at least a day in rats exhibiting ketosis and hypoglycaemia, could be due to a functional defect in hepatic gluconeogenesis. Furthermore, KD rats had lower levels of insulin (Gumus et al. 2022). Glucose synthesis is essential for brain and cellular energy (Rask‐Madsen and Kahn 2012). Studies show that rodents on KD (60% fat, 20% protein, 20% carbohydrate) develop impaired glucose tolerance and disrupted insulin signaling within 2 days (Lv et al. 2023; Wiedemann et al. 2013). In addition, a study by Grandl et al. (2018) showed that, even though KD fed animals appear to be healthy in the fasted state, they exhibit decreased glucose tolerance as compared to control groups (Long et al. 2023).

On the other hand, the reduction in TG and FFA level noticed in rat group fed KD resulted from breaking down of TG into glycerol and fatty acids, and transforming of fatty acids into ketone bodies via β‐oxidation in hepatocytes (Dankel et al. 2023). Ketone bodies can be produced not only during fasting but also due to disruption of carbohydrate metabolism (López‐Ojeda and Hurley 2023). These findings are consistent with those reported by earlier studies (Dell et al. 2001; Dustin and Stafstrom 2016; Taha et al. 2005). Furthermore, evidence was provided by Kume et al. (2007) that, the metabolism of serum lipids in mice was modified because of the KD, particularly in terms of the lipogenesis‐lipolysis equilibrium, causing lipids accumulation in kidneys, liver, and serum (Kundu et al. 2022). Meanwhile, other research found that the levels of total cholesterol and TG in plasma were markedly increased by a KD (Kwiterovich et al. 2003; Meidenbauer et al. 2014).

KD rats displayed markedly higher levels of ketone bodies and lactate. β‐hydroxybutyrate and acetoacetate are the primary ketone bodies (Hasanpour et al. 2020). Ketosis is the state associated with high ketone body levels in the blood and has a therapeutic action in a number of medical conditions (Oyindasola 2023). Beyond systemic metabolism, recent evidence suggests a synergistic interaction between bone marrow adipose tissue and KD. BMAT provides a specialized lipid reservoir that supports ketogenesis under carbohydrate restriction, thereby amplifying ketone body production and sustaining energy supply. However, this synergy may also compromise skeletal integrity, as KD‐induced acidosis and mineral imbalance can accelerate bone turnover and reduce bone density (Dzubanova et al. 2024; Garofalo et al. 2023; Merlotti et al. 2021). Excess ketone bodies from KD lower bicarbonate reserves, causing blood pH to drop and ketoacidosis to develop (Weber et al. 2020).

In insulin resistance, excess fatty acids reach tissues, driving ketogenesis and overproduction of ketone bodies, which can lead to diabetic ketoacidosis (Patel 2023). KD caused hypoglycemia due to depleted liver glycogen, unsuitable for high‐intensity exercise (Burke 2015; Fukazawa et al. 2020). KD rats showed doubled lactate and reduced gluconeogenic enzymes (PEPCK, pyruvate carboxylase) (Tripathy 2023). BM's diverse compounds suggest therapeutic potential in diabetes (Sharma and Kumar 2023).

Yuan et al. (2008) showed BM peptide lowered blood glucose in diabetic mice, while TG and cholesterol findings indicated BM prevented lipid accumulation, linking glucose regulation with improved lipid metabolism (Liu et al. 2015). Similar results were reported by Bai et al. (2016) and Jiang et al. (2016).

Yousaf et al. (2016) showed BM enhances insulin via β‐cell repair. Yu et al. (2013) found BM sterols prevent intestinal cholesterol absorption, producing hypocholesterolemic effects, though TG reduction was limited (Huang et al. 2008) confirmed similar results in cholesterol‐fed rats. Consistently, Senanayake et al. (2004) reported BM powder lowered serum and liver lipids, especially TG, indicating BM compounds attenuate lipid disorders such as hyperlipidemia and fatty liver. BM contains saponins (cucurbit, oleanane, ursulan, stigmasterol, cholesterol, sitosterol) that lower visceral fat, glucose, and TG by enhancing liver and adipose oxidation (Fan et al. 2019). Gao et al. (2018) showed BM polysaccharides modulate lipid metabolism, reducing cholesterol and TG via diosgenin, fiber, and phytosterols. Saad et al. (2017) reported BM reduces body weight and fat accumulation, while Mahwish et al. (2017) confirmed anti‐hyperglycemic and anti‐hyperlipidemic effects in T2D. Akhter et al. (2018) further noted BM extract decreased high TG levels in diabetic rats. BM improved glucose levels via insulin‐mimetic compounds, AMPK activation, β‐cell protection, and antioxidant effects, as supported by recent studies (Chang 2025; Richter et al. 2023).

BM contains phenols, flavonoids, terpenes, glucosinolates, saponins, alkaloids, minerals, and fiber, contributing to its antioxidant and therapeutic potential (Sethi and Dahiya 2019). Studies show BM influences glycolysis/gluconeogenesis and ketone metabolism (Hasanpour et al. 2020), improves fat metabolism and reduces dyslipidemia in T2D patients (Kumari et al. 2018), and BM saponins lower elevated ketone bodies in obese rats (Liu et al. 2018; Zhao et al. 2020). BM supplementation improved lipid profile, lowering triglycerides, free fatty acids, and cholesterol, thereby reducing cardiovascular risk and supporting its role as a safe adjunct to KD (Amini et al. 2024). Virdi et al. (2003) and Ahmad et al. (2012) showed BM extract elevated liver glycogen by improving glucose intolerance. Under KD‐induced low glucose, BM also enhanced gluconeogenesis via increased PEPCK activity, promoting glucose production from lactate and citric acid cycle precursors (Sasaki et al. 2017). Similarly, Lin et al. (2020) showed BM extract reduced hepatic pyruvate carboxylase, while Chen and Li (2003) reported that BM juice improved oral glucose tolerance, lowered ketone bodies, and reduced visceral fat in high‐fat diet rats.

## 5 Conclusions

BM extract in KD rats normalized insulin and glucose, reduced TG, cholesterol, and ketone bodies, supporting regulation of gluconeogenesis and ketogenesis. Limitations include short study duration, lack of extract standardization, unassessed molecular mechanisms, use of only male rats, and unquantified compound contributions. Future work should explore long‐term, mechanistic, and sex‐specific effects.

## Author Contributions

**Ghalia Shamlan:** conceptualization, investigation, funding acquisition, writing – original draft, writing – review and editing, visualization, validation, methodology, software, formal analysis, project administration, resources, supervision, data curation.

## Funding

This work was supported by the ongoing research funding program (ORF‐2025‐631), King Saud University, Riyadh, Saudi Arabia.

## Ethics Statement

The study was conducted in accordance with the Declaration of Helsinki, and approved by Standing Committee for Scientific Research (REC‐45/07/968).

## Conflicts of Interest

The author declares no conflicts of interest.

## Data Availability

All data included in this study are available from the corresponding author upon reasonable request.
